# Efficient quantum circuits for dense circulant and circulant like operators

**DOI:** 10.1098/rsos.160906

**Published:** 2017-05-10

**Authors:** S. S. Zhou, J. B. Wang

**Affiliations:** 1 Kuang Yaming Honors School, Nanjing University, Nanjing 210093, People’s Republic of China; 2 Department of Physics, Yale Quantum Institute, Yale University, New Haven, CT 06520, USA; 3 Department of Applied Physics, Yale Quantum Institute, Yale University, New Haven, CT 06520, USA; 4 School of Physics, The University of Western Australia, Perth, Western Australia 6009, Australia

**Keywords:** quantum computation, quantum circuit, dense circulant operator, block circulant operator, Toeplitz and Hankel matrices

## Abstract

Circulant matrices are an important family of operators, which have a wide range of applications in science and engineering-related fields. They are, in general, non-sparse and non-unitary. In this paper, we present efficient quantum circuits to implement circulant operators using fewer resources and with lower complexity than existing methods. Moreover, our quantum circuits can be readily extended to the implementation of Toeplitz, Hankel and block circulant matrices. Efficient quantum algorithms to implement the inverses and products of circulant operators are also provided, and an example application in solving the equation of motion for cyclic systems is discussed.

## 1. Introduction

Quantum computation exploits the intrinsic nature of quantum systems in a way that promises to solve problems otherwise intractable on conventional computers. As the most widely used model of quantum computation, a quantum circuit provides a complete description of a specified quantum algorithm, whose computational complexity is determined by the number of quantum gates required. In general, the number of two-level gates (i.e. unitary matrices acting non-trivially on two-dimensional subspaces, which are universal for computation) needed to decompose an arbitrary unitary in *N* dimensions scales as *O*(*N*^2^). There are many known *N*-dimensional matrices that cannot be decomposed as a product of fewer than *N*−1 two-level gates [1], and thus cannot be implemented efficiently on a quantum computer. An essential research focus in quantum computation is to explore which kinds of linear operations (either unitary or non-unitary) can be efficiently implemented using O(poly(log⁡N)) number of elementary quantum gates (i.e. one- or two-level unitary matrices) and measurements.

Significant breakthroughs in the area include the development of efficient quantum algorithms for Hamiltonian simulation, which is central to the studies of chemical and biological processes [2–8]. Recently, Berry, Childs and Kothari presented an algorithm for sparse Hamiltonian simulation achieving near-linear scaling with the sparsity and sublogarithmic scaling with the inverse of the error [8]. Additionally, using the Hamiltonian simulation algorithm as an essential ingredient, Harrow *et al.* [9] showed that for a sparse and well-conditioned matrix *A*, there is an efficient algorithm (known as the HHL algorithm) that provides a quantum state proportional to the solution $\sum_{j}x_{j}\left|j\right\rangle$ of the linear system of equations *A****x***=***b***.

However, as proven by Childs & Kothari [10], it is impossible to perform a generic simulation of an arbitrary dense Hamiltonian *H* in $C^{N\times N}$ in time O(poly(∥H∥,log⁡N)), where ∥*H*∥ is the spectral norm, but possible for certain non-trivial classes of Hamiltonians. It is then natural to ask under what conditions we can extend the sparse Hamiltonian simulation algorithm and the HHL algorithm to the realm of dense matrices. In this paper, we use the ‘unitary decomposition’ approach developed by Berry *et al.* [7] to implement dense circulant Hamiltonians in time O(poly(∥H∥,log⁡N)). Combining this with the HHL algorithm, we can also efficiently implement the inverse of dense circulant matrices and thus solve systems of circulant matrix linear equations.

Furthermore, we provide an efficient algorithm to implement circulant matrices *C* directly, by decomposing them into a linear combination of unitary matrices. We then apply the same technique to implement block circulant matrices, Toeplitz and Hankel matrices, which have significant applications in physics, mathematics and engineering [11–21]. For example, we can simulate classical random walks on circulant, Toeplitz and Hankel graphs [22,23]. In fact, any arbitrary matrix can be decomposed into a product of Toeplitz matrices [24]. If the number of Toeplitz matrices required is in the order of O(poly(log⁡N)), we can have an efficient quantum circuit.

This paper is organized as follows. In §2, we present an algorithm to implement circulant matrices, followed by discussions on block circulant matrices, Toeplitz and Hankel matrices in §3. In §§4 and 5, we provide efficient methods to simulate circulant Hamiltonians and to implement the inverse of circulant matrices. In §6, we describe a technique to efficiently implement products of circulant matrices. In the last section, we provide an example application in solving the equation of motion for vibrating systems with cyclic symmetry.

## 2. Implementation of circulant matrices

A circulant matrix has each row right-rotated by one element with respect to the previous row, defined as

2.1 $$C=\left(\begin{array}{cccc} c_{0} & c_{1} & \cdots & c_{N-1}\\ c_{N-1} & c_{0} & \cdots & c_{N-2}\\ \vdots & \vdots & \ddots & \vdots \\ c_{1} & c_{2} & \cdots & c_{0} \end{array}\right),$$

using an *N*-dimensional vector ***c***=(*c*_0_ *c*_1_ ⋯ *c*_*N*−1_) [25]. In this paper, we will assume *c*_*j*_ to be non-negative for all *j*, which is often the case in practical applications. We also assume that the spectral norm (the largest eigenvalue) $∥C∥=\sum_{j=0}^{N-1}c_{j}$ of the circulant matrix *C* is equal to 1 for simplicity.

Note that *C* can be decomposed into a linear combination of efficiently realizable unitary matrices as follows:

2.2 $$C=\left(\begin{array}{cccc} c_{0} & c_{1} & \cdots & c_{N-1}\\ c_{N-1} & c_{0} & \cdots & c_{N-2}\\ \vdots & \vdots & \ddots & \vdots \\ c_{1} & c_{2} & \cdots & c_{0} \end{array}\right)=c_{0}\left(\begin{array}{cccc} 1 & 0 & \cdots & 0\\ 0 & 1 & \cdots & 0\\ \vdots & \vdots & \ddots & \vdots \\ 0 & 0 & \cdots & 1 \end{array}\right)+c_{1}\left(\begin{array}{cccc} 0 & 1 & \cdots & 0\\ 0 & 0 & \cdots & \vdots \\ \vdots & \vdots & \ddots & 1\\ 1 & 0 & \cdots & 0 \end{array}\right)+\cdots =\sum_{j=0}^{N-1}c_{j}V_{j},$$

where $V_{j}=\sum_{k=0}^{N-1}\left|(k-j)\;\mathrm{mod}\;\;N\right\rangle \left\langle k\right|$. Such a linear combination of unitary matrices can be dealt with by the unitary decomposition approach introduced by Berry *et al.* [7]. For completeness, we restate their method as lemma 2.1 given below.

**Lemma 2.1.**

*Let* $M=\sum_{j=0}^{J}\alpha_{j}W_{j}$ *be a linear combination of unitaries W*_*j*_ *with α*_*j*_≥0 *for all j and* $\sum_{j=0}^{J}\alpha_{j}=1$. *Let O*_*α*_ *be any operator that satisfies* $O_{\alpha }\left|0^{m}\right\rangle =\sum_{j=0}^{J}\sqrt{\alpha_{j}}\left|j\right\rangle$, *where m is the number of qubits used to represent* |*j*〉, *and* $\mathrm{select}(W)=\sum_{j=0}^{J}\left|j\right\rangle \left\langle j\right|⊗W_{j}$. *Then*

2.3 $$(O_{\alpha }^{†}⊗I)\;\mathrm{select}(W)(O_{\alpha }⊗I)\left|0^{m}\right\rangle \left|\psi \right\rangle =\left|0^{m}\right\rangle M\left|\psi \right\rangle +\left|\Psi^{⊥}\right\rangle ,$$

*where* (|0^*m*^〉〈0^*m*^|⊗*I*)|*Ψ*^⊥^〉=0.

**Figure 1.**
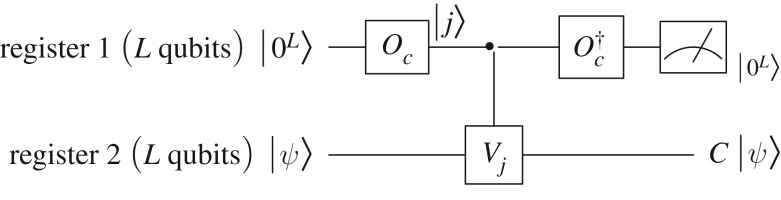
Quantum circuit to implement a circulant matrix.

Unless stated otherwise, we assume that *N*=2^*L*^, where *L* is an integer. If *N* is not a power of two, we will need to embed the system into a larger Hilbert space whose dimension is a power of two. On the other hand, it is also convenient to simply discretize practical problems using powers of two. Lemma 2.1 can be directly applied to implement the circulant matrix *C*, as shown in figure 1, by taking *M*=*C*, *α*_*j*_=*c*_*j*_, *W*_*j*_=*V*_*j*_, *J*=2^*L*^ and *m*=*L*. Since select(*V*)|*j*〉|*k*〉=|*j*〉|(*k*−*j*) mod  *N*〉, it can be implemented using quantum adders [26,27], which requires O(log⁡N) one- or two-qubit gates. Note that when *N* is not a power of two, it may take additional O(log⁡N) ancillary qubits to implement the ‘*mod* *N*’ operation in select(*V*), for example, by first subtracting *N* from *k*−*j* and then using the sign qubit to control the ‘*mod* *N*’ operation.

A measurement result of |0^*L*^〉 in the first register generates the required state *C*|*ψ*〉 in the second register. The probability of this measurement outcome is *O*(∥*C*|*ψ*〉∥^2^). With the help of amplitude amplification [28], this can be further improved, requiring only *O*(1/∥*C*|*ψ*〉∥) rounds of application of $\left(O_{c}^{†}⊗I)\;\mathrm{select}(V)(O_{c}⊗I\right)$. The amplitude amplification procedure also requires the same number of applications of *O*_*ψ*_, where *O*_*ψ*_|0^*L*^〉=|*ψ*〉, and its inverse in order to reflect quantum states about the initial state |0^*L*^〉|*ψ*〉. If *O*_*ψ*_ is unknown, amplitude amplification is not applicable and we will need to repeat the measuring process in figure 1 *O*(1/∥*C*|*ψ*〉∥^2^) times, during which *O*(1/∥*C*|*ψ*〉∥^2^) copies of |*ψ*〉 are required. It is worth noting that with the assumption *c*_*j*_≥0, *C* is unitary if and only if *C*=*V*_*j*_. In other words, a non-trivial circulant matrix is non-unitary and therefore, the oblivious amplitude amplification procedure [29] cannot be applied.

Provided with the oracle *O*_*c*_ satisfying $O_{c}\left|0^{L}\right\rangle =\sum_{j=0}^{N-1}\sqrt{c_{j}}\left|j\right\rangle$, theorem 2.2 follows directly from the above discussions. *O*_*c*_ can be efficiently implemented for certain efficiently computable vectors ***c*** [30–32]. Another way to construct states like $\sum_{j=0}^{N-1}\sqrt{c_{j}}\left|j\right\rangle$ is via qRAM, which uses *O*(*N*) hardware resources but only O(log⁡N) operations to access them [33,34].

**Theorem 2.2 (Implementation of circulant matrices).**

*There exists an algorithm creating the quantum state C*|*ψ*〉 *for an arbitrary quantum state* $\left|\psi \right\rangle =\sum_{k=0}^{N-1}\psi_{k}\left|k\right\rangle$, *using O*(1/∥*C*|*ψ*〉∥) *calls of O*_*c*_*, O*_*ψ*_ *and their inverses, as well as* O(log⁡N/∥C|ψ⟩∥) *additional one- or two-qubit gates.*

The complexity in theorem 2.2 is inversely proportional to the square root of *p*=∥*C*|*ψ*〉∥^2^, which depends on the quantum state to be acted upon. Specifically, |*C*|*ψ*〉|^2^=〈*ψ*∥*C*^†^*C*∥*ψ*〉=〈*ψ*|*FΛ*^†^*F*^†^*FΛF*^†^|*ψ*〉=〈*ψ*|*FΛ*^†^*ΛF*^†^|*ψ*〉. Here we use the diagonal form of *C* [25], *C*=*FΛF*^†^, where *F* is the Fourier matrix with $F_{kj}=e^{2\pi ijk/N}/\sqrt{N}$ and *Λ* is a diagonal matrix of eigenvalues given by $\Lambda_{k}=\sum_{j=0}^{N-1}c_{j}\;e^{2\pi ijk/N}$. Since the spectral norm ∥*C*∥ of the circulant matrix *C* is equal to one, we have *p*=〈*ψ*|*FΛ*^†^*ΛF*^†^|*ψ*〉≥1/*κ*^2^, where *κ* is the condition number, defined as the ratio between *C*’s largest and smallest (absolute value of) eigenvalues [9]. Therefore, our algorithm is bound to perform well when κ=O(poly(log⁡N)). In the ideal case where *κ*=1 and *p*=1, the vector ***c*** is a unit basis in which only one element is equal to one and the others are zero.

Even when *κ* is large, our algorithm is still efficient when the input quantum state after Fourier transform is in the subspace whose corresponding eigenvalues are large. For example, when $\Lambda_{k}=\cos(2\pi k/N)$ we have κ=∞ when *N*>2. $p=\left\langle \phi \right|\Lambda^{†}\Lambda \left|\phi \right\rangle =\sum_{k=0}^{N-1}\cos{\left(2\pi k/N\right)}^{2}|\phi_{k}{|}^{2}\geq \sum_{k\notin [N/8,3N/8]\cup [5N/8,7N/8]}\frac{1}{2}|\phi_{k}{|}^{2}$ in which $\left|\phi \right\rangle :=F^{†}\left|\psi \right\rangle =\sum_{k=0}^{N-1}\phi_{k}\left|k\right\rangle$. The success rate is therefore lower-bounded by a constant as long as the input quantum state is restricted in a subspace such that *ϕ*_*k*_=0 when *k*∈[*N*/8,3*N*/8]∪[5*N*/8,7*N*/8].

## 3. Circulant-like matrices

### 3.1. Block circulant matrices

Some block circulant matrices with special structures can also be implemented efficiently in a similar manner. We assume the blocks are *N*′-dimensional matrices and $L^{\prime }=\log N^{\prime }$ in the following discussions.

Firstly, when each block is a unitary operator up to a constant factor (i.e. $C_{j}=c_{j}U_{j}$), we have a unitary block (UB) matrix:

3.1 $$\begin{array}{rl} C_{UB}=\left(\begin{array}{cccc} C_{0} & C_{1} & \cdots & C_{N-1}\\ C_{N-1} & C_{0} & \cdots & C_{N-2}\\ \vdots & \vdots & \ddots & \vdots \\ C_{1} & C_{2} & \cdots & C_{0} \end{array}\right) & =\left(\begin{array}{cccc} 1 & 0 & \cdots & 0\\ 0 & 1 & \cdots & 0\\ \vdots & \vdots & \ddots & \vdots \\ 0 & 0 & \cdots & 1 \end{array}\right)⊗C_{0}+\left(\begin{array}{cccc} 0 & 1 & \cdots & 0\\ 0 & 0 & \cdots & \vdots \\ \vdots & \vdots & \ddots & 1\\ 1 & 0 & \cdots & 0 \end{array}\right)⊗C_{1}+\cdots \\ & =\sum_{j=0}^{N-1}V_{j}⊗C_{j}=\sum_{j=0}^{N-1}c_{j}V_{j}⊗U_{j}. \end{array}$$

**Figure 2.**
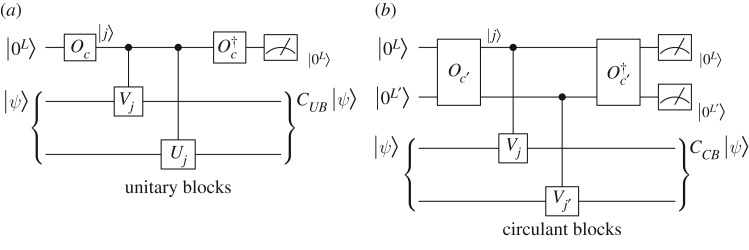
The quantum circuit to implement block circulant matrices with special structures.

If the set of blocks ${\left\{U_{j}\right\}}_{j=0}^{N-1}$ can be efficiently implemented, then by simply replacing $\mathrm{select}(V)=\sum_{j=0}^{N-1}\left|j\right\rangle \left\langle j\right|⊗V_{j}$ with $\sum_{j=0}^{N-1}\left|j\right\rangle \left\langle j\right|(V_{j}⊗U_{j})$, we can efficiently implement the block circulant matrices *C*_*UB*_ using the same algorithm discussed in §2 as illustrated in figure 2*a*.

Specifically, when the set of blocks ${\left\{U_{j}\right\}}_{j=0}^{N-1}$ are one-dimensional, we can implement complex-valued circulant matrices with efficiently computable phase. For example, for $U_{j}=(e^{i\theta j})$, *j*=0,1,…,*N*−1, circulant matrices with the parameter vector ***c***=(*c*_0_,*e*^*iθ*^*c*_1_,…, e^i(*N*−1)*θ*^*c*_*N*−1_) can be implemented efficiently. Moreover, if *θ*=*π*, ***c***=(*c*_0_,−*c*_1_,…,(−1)^*N*−1^ *c*_*N*−1_) corresponding to the circulant matrix with negative elements on odd-numbered sites is efficiently implementable.

Another important family is block circulant matrices with circulant blocks (CB), which has found a wide range of applications in algorithms, mathematics, etc. [18–21]. It is defined as follows:

3.2 $$C_{CB}=\left(\begin{array}{cccc} C_{0} & C_{1} & \cdots & C_{N-1}\\ C_{N-1} & C_{0} & \cdots & C_{N-2}\\ \vdots & \vdots & \ddots & \vdots \\ C_{1} & C_{2} & \cdots & C_{0} \end{array}\right),$$

where $C_{j}$ is a circulant matrix specified by a *N*′-dimensional vector ***c***_*j*_=(*c*_*j*0_ *c*_*j*1_ ⋯ *c*_*j*(*N*′−1)_). *C*_*CB*_ is a *N*×*N*′-dimensional matrix determined by *N*×*N*′ parameters {*c*_*jj*′_}_*j*=0,…,*N*−1 *j*′=0,…,*N*′−1_. It can be decomposed as follows:

3.3 $$C_{CB}=\sum_{j=0}^{N-1}\sum_{j^{\prime }=0}^{N^{\prime }-1}c_{jj^{\prime }}V_{j}⊗V_{j^{\prime }}.$$

Given an oracle *O*_*c*′_ satisfying $O_{c^{\prime }}\left|0^{L+L^{\prime }}\right\rangle =\sum_{j=0}^{N-1}\sum_{j=0}^{N^{\prime }-1}c_{jj^{\prime }}\left|j\right\rangle \left|j^{\prime }\right\rangle$, we can implement *C*_*CB*_ using the quantum circuit shown in figure 2*b*, which adopts a combination of two quantum subtractors.

### 3.2. Toeplitz and Hankel matrices

A Toeplitz matrix is a matrix in which each descending diagonal from left to right is constant, which can be written explicitly as

3.4 $$T=\left(\begin{array}{ccccc} t_{0} & t_{-1} & \cdots & t_{-(N-2)} & t_{-(N-1)}\\ t_{1} & t_{0} & \cdots & t_{-(N-3)} & t_{-(N-2)}\\ t_{2} & t_{1} & \cdots & t_{-(N-4)} & t_{-(N-3)}\\ \vdots & \vdots & \ddots & \vdots & \vdots \\ t_{N-1} & t_{N-2} & \cdots & t_{1} & t_{0} \end{array}\right),$$

specified by 2*N*−1 parameters. We focus on the situation where *t*_*j*_≥0 for all *j* as in §2. Clearly, when *t*_−(*N*−*i*)_=*t*_*i*_ for all *i*, *T* is a circulant matrix. Although a Toeplitz matrix is not circulant in general, any Toeplitz matrix *T* can be embedded in a circulant matrix [15,35], defined by

3.5 $$C_{T}=\left(\begin{array}{cc} T & B_{T}\\ B_{T} & T \end{array}\right),$$

where *B*_*T*_ is another Toeplitz matrix defined by

3.6 $$B_{T}=\left(\begin{array}{ccccc} 0 & t_{N-1} & \cdots & t_{2} & t_{1}\\ t_{-(N-1)} & 0 & \cdots & t_{3} & t_{2}\\ t_{-(N-2)} & t_{-(N-1)} & \cdots & t_{4} & t_{3}\\ \vdots & \vdots & \ddots & \vdots & \vdots \\ t_{-1} & t_{-2} & \cdots & t_{-(N-1)} & 0 \end{array}\right).$$

As a result, we use this embedding to implement Toeplitz matrices because

3.7 $$\left(\begin{array}{cc} T & B_{T}\\ B_{T} & T \end{array}\right)\left(\begin{array}{c} \psi \\ 0 \end{array}\right)=\left(\begin{array}{c} T\psi \\ B_{T}\psi \end{array}\right).$$

**Figure 3.**
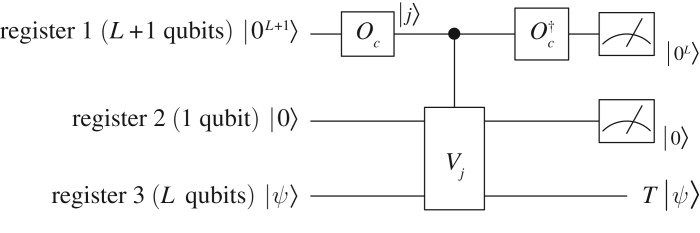
The quantum circuit to implement a Toeplitz matrix. In this figure, $O_{c}\left|0^{L+1}\right\rangle =\sum_{j=0}^{2N-1}c_{j}\left|j\right\rangle$, where ***c***=(*t*_0_ *t*_−1_⋯*t*_−(*N*−1)_ 0 *t*_*N*−1_⋯*t*_1_).

Therefore, by implementing *C*_*T*_, we obtain a quantum state proportional to |0〉*T*|*ψ*〉+|1〉*B*_*T*_|*ψ*〉. Then we do a quantum measurement on the single qubit (in the second register in figure 3) to obtain the quantum state *T*|*ψ*〉. The success rate is ∥*T*|*ψ*〉∥^2^ according to theorem 2.2 under the normalization condition that $\sum_{j=-(N-1)}^{N-1}t_{j}=\sum_{j=0}^{N-1}c_{j}=1$. With the help of amplitude amplification, only *O*(1/∥*T*|*ψ*〉∥) applications of the circuit in figure 3 are required.

A Hankel matrix is a square matrix in which each ascending skew-diagonal from left to right is constant, which can be written explicitly as

3.8 $$H=\left(\begin{array}{ccccc} h_{N-1} & h_{N-2} & \cdots & h_{1} & h_{0}\\ h_{N-2} & h_{N-3} & \cdots & h_{0} & h_{-1}\\ \vdots & \vdots & \ddots & \vdots & \vdots \\ h_{1} & h_{0} & \cdots & h_{-(N-3)} & h_{-(N-2)}\\ h_{0} & h_{-1} & \cdots & h_{-(N-2)} & h_{-(N-1)} \end{array}\right),$$

specified by 2*N*−1 non-negative parameters. A permutation matrix $P=\sigma_{x}^{⊗L}$ transforms a Hankel matrix into a Toeplitz matrix. It can be easily verified that *T*=*HP* and *H*=*TP*, in which *t*_*j*_=*h*_*j*_ for all *j*. Note that when *N* is not a power of two, we need to be careful with the embedding when mapping a circulant matrix into a Hankel matrix. The subspace *span*{|0〉,…,|*N*−1〉} in the implementation of circulant matrices corresponds to the subspace *span*{|2^*L*^−*N*〉,…,|2^*L*^−1〉} in the implementation of Hankel matrices.

Therefore by inserting the permutation *P* before the implementation of *T*, the circuit in figure 3 can be used to implement *H*, and the success rate is ∥*H*|*ψ*〉∥^2^ under the normalization condition that $\sum_{j=-(N-1)}^{N-1}h_{j}=\sum_{j=0}^{N-1}c_{j}=1$. With the help of amplitude amplification, only *O*(1/∥*H*|*ψ*〉∥) applications are required.

In comparison with existing algorithms, such as that described in [35], the above described quantum circuit provides a better way to realize circulant-like matrices, requiring fewer resources and with lower complexity. For example, only 2log⁡N qubits are required to implement *N*-dimensional Toeplitz matrices, which is a significant improvement over the algorithm presented in [35] via sparse Hamiltonian simulations. More importantly, this is an exact method and its complexity does not depend on an error term. It is also not limited to sparse circulant matrices *C* as in [35]. Moreover, implementation of non-unitary matrices, such as circulant matrices, is not only of importance in quantum computing, but also a significant ingredient in quantum channel simulators [36,37], because the set of Kraus operators in the quantum channel $\rho \mapsto \sum_{i}K_{i}\rho K_{i}^{†}$ is normally non-unitary [1]. The simplicity of our circuit increases its feasibility in experimental realizations.

## 4. Circulant Hamiltonians

Hamiltonian simulation is expected to be one of the most important undertakings for quantum computation. It is therefore important to explore the possibility of efficient implementation of circulant Hamiltonians because of their extensive applications. Particularly, the implementation of e^−iCt^ is equivalent to the implementation of continuous-time quantum walks on a weighted circulant graph [38,39]. Moreover, simulation of Hamiltonians is also an important part in the HHL algorithm to solve linear systems of equations [9].

A number of algorithms have been shown to be able to efficiently simulate sparse Hamiltonians [2–8], including the unitary decomposition approach [7]. We show that this approach can be extended to the simulation of dense circulant Hamiltonians. It is well known that circulant matrices are diagonalizable as e^−iCt^=*F* *e*^−i*Λ*t^*F*^†^. In general, implementing an arbitrary diagonal unitary requires up to O(Nlog⁡N) one- or two-qubit gates [40]. However, when ${\left\{\Lambda_{k}\right\}}_{k=0}^{N-1}$ can be efficiently computed, one can efficiently implement e^−iCt^ [23,41,42].

In this section, we will focus on the simulation of Hermitian circulant matrices, when e^−iCt^ is unitary. For completeness, we first summarize briefly the unitary decomposition approach in [7] and then discuss how it can be used to efficiently simulate dense circulant Hamiltonians. To simulate *U*=e^−iCt^, the evolution time *t* is divided into *r* segments with *U*_*r*_=e^−iCt/*r*^, which can be approximated as $\tilde{U}=\sum_{k=0}^{K}1/k!{\left(-\mathrm{iCt}/r\right)}^{k}$ with error *ϵ*. It can be proven that if we choose K=O(log⁡(r/ϵ)/loglog⁡(r/ϵ))=O(log⁡(t/ϵ)/loglog⁡(t/ϵ)), then $∥U_{r}-\tilde{U}∥\leq \epsilon /r$ and the total error is within *ϵ*.

Since $C=\sum_{j=0}^{N-1}c_{j}V_{j}$ as given by equation (2.2), we have

4.1 $$\tilde{U}=\sum_{k=0}^{K}\frac{{\left(-\mathrm{iCt}/r\right)}^{k}}{k!}=\sum_{k=0}^{K}\sum_{j_{1},\ldots ,j_{k}=0}^{N-1}\frac{{\left(-it/r\right)}^{k}}{k!}c_{j_{1}}\cdots c_{j_{k}}V_{j_{1}}\cdots V_{j_{k}}.$$

Let *W*_(*k*,*j*_1_,…,*j*_*k*_)_=(−*i*)^*k*^*V*_*j*_1__⋯*V*_*j*_*k*__ and

4.2 $$O_{\alpha }\left|0^{K+KL}\right\rangle =\frac{1}{\sqrt{s}}\sum_{k=0}^{K}\sum_{j_{1},\ldots ,j_{k}=0}^{N-1}\sqrt{{\left(t/r\right)}^{k}/k!c_{j_{1}}\cdots c_{j_{k}}}\left|1^{k}0^{K-k}\right\rangle \left|j_{1}\right\rangle \cdots \left|j_{k}\right\rangle \left|0^{(K-k)L}\right\rangle ,$$

where |1^*k*^0^*K*−*k*^〉 is the unary encoding of *k*. Here, *s* is the normalization coefficient and we choose r=⌈t/ln⁡2⌉ so that

4.3 $$s=\sum_{k=0}^{K}\sum_{j_{1},\ldots ,j_{k}=0}^{N-1}\frac{{\left(t/r\right)}^{k}}{k!}c_{j_{1}}\cdots c_{j_{k}}=\sum_{k=0}^{K}\frac{{\left((c_{0}+\cdots +c_{N-1})t/r\right)}^{k}}{k!}\approx 2.$$

By taking $M=\tilde{U}$, $\alpha_{j}=\sqrt{{\left(t/r\right)}^{k}/k!c_{j_{1}}\cdots c_{j_{k}}}$, *W*_*j*_=*W*_(*k*,*j*_1_,…,*j*_*k*_)_, *J*=*KN*^*K*^ and *m*=*K*+*KL* in lemma 2.1, we have

**Figure 4.**
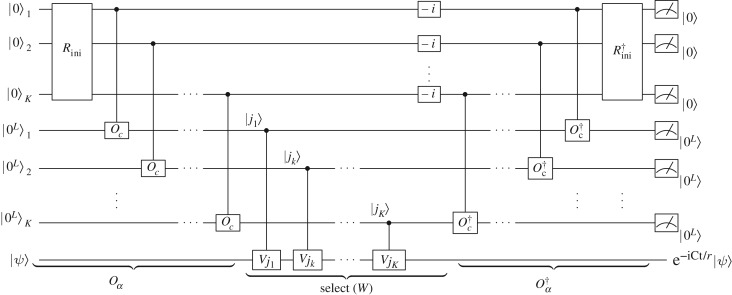
The quantum circuit to implement one segment of circulant Hamiltonians. Here Rini|0K⟩=∑k=0K(t/r)k/k!|1k0K−k⟩ and −i=|0⟩⟨0|+(−i)|1⟩⟨1|.

4.4 $$(O_{\alpha }^{†}⊗I)\mathrm{select}(W)(O_{\alpha }⊗I)\left|0^{K+KL}\right\rangle \left|\psi \right\rangle =\frac{1}{s}\left|0^{K+KL}\right\rangle \tilde{U}\left|\psi \right\rangle +\left|\Psi^{⊥}\right\rangle ,$$

where (|0^*K*+*KL*^〉〈0^*K*+*KL*^|⊗*I*)|*Ψ*^⊥^〉=0. It has been shown in [7] that after one step of oblivious amplitude amplification procedure [29], *U*_*r*_=e^−iCt/*r*^ can be simulated within error *ϵ*/*r*. The oblivious amplitude amplification procedure avoids the repeated preparations of |*ψ*〉 so that $\tilde{U}\left|\psi \right\rangle$ can be obtained using only one copy of |*ψ*〉, as shown in figure 4. The total complexity depends on the number of gates required to implement select(*W*) and *O*_*α*_.

If *C* is not Hermitian (and $\tilde{U}$ is not at least approximately unitary), the oblivious amplitude amplification procedure [29] will not be applicable, and then we have to resort to the traditional amplitude amplification [28]. This will lead to a complexity depending exponentially on *t* because we have to run the amplitude amplification recursively, but the complexity will still depend logarithmically on *N*.

**Theorem 4.1 (Simulation of circulant Hamiltonians).**

*There exists an algorithm performing e*^−iCt^ *on an arbitrary quantum state |ψ〉 within error ϵ, using* O(t(log⁡(t/ϵ)/loglog⁡(t/ϵ))) *calls of controlled-O*_*c*_^1^ *and its inverse, as well as* O(t(log⁡N)(log⁡(t/ϵ)/loglog⁡(t/ϵ))) *additional one- and two-qubit gates.*

**Proof.**

We first consider the number of gates used to implement *O*_*α*_ in equation (4.2). It can be decomposed into two steps. The first step is to create the normalized version of the state $\sum_{k=0}^{K}\sqrt{{\left(t/r\right)}^{k}/k!}\left|1^{k}0^{K-k}\right\rangle$ from the initial state |0^*K*^〉, which takes *O*(*K*) consecutive one-qubit rotations on each qubit. We then apply *K* sets of controlled-*O*_*c*_ to transform |0^*L*^〉 into $\sum_{j=0}^{N-1}\sqrt{c_{j}}\left|j\right\rangle$ when the control qubit is |1〉. We therefore need *O*(*K*) calls of controlled-*O*_*c*_ and *O*(*K*) additional one-qubit gates to implement *O*_*α*_.

Next we focus on the implementation of

4.5 $$\begin{array}{rl} & \mathrm{select}(W)=\sum_{\left(k,j_{1},j_{2},\cdots ,j_{k}\right)}\\ & \;\left|1^{k}0^{K-k}\right\rangle \left|j_{1}\right\rangle \cdots \left|j_{k}\right\rangle \left|0^{(K-k)L}\right\rangle \left\langle 1^{k}0^{K-k}\right|\left\langle j_{1}\right|\cdots \left\langle j_{k}\right|\left\langle 0^{(K-k)L}\right|⊗{\left(-i\right)}^{k}V_{j_{1}}\cdots V_{j_{k}}, \end{array}$$

which performs the transformation

4.6 $$\begin{array}{rl} & \left|1^{k}0^{K-k}\right\rangle \left|j_{1}\right\rangle \cdots \left|j_{k}\right\rangle \left|0^{(K-k)L}\right\rangle \left|\psi \right\rangle \overset{\text{select}(W)}{\to }\\ & \;\left|1^{k}0^{K-k}\right\rangle \left|j_{1}\right\rangle \cdots \left|j_{k}\right\rangle \left|0^{(K-k)L}\right\rangle {\left(-i\right)}^{k}V_{j_{1}}\cdots V_{j_{k}}\left|\psi \right\rangle . \end{array}$$

As *V*_*j*_|ℓ〉=|(ℓ−*j*) mod  *N*〉, we can transform |*ψ*〉 into *V*_*j*_1__⋯*V*_*j*_*k*__|*ψ*〉 by applying *K* quantum subtractors between |*j*〉_*j*=*j*_1_,*j*_2_,…,*j*_*k*_,0,…_ and |*ψ*〉. *K* phase gates on each of the first *K* qubits multiply the amplitude by (−*i*)^*k*^. Therefore, select(*W*) can be decomposed into O(Klog⁡N) one or two-qubit gates.

In summary, *O*(*K*) calls of controlled-*O*_*c*_ and its inverse as well as O(Klog⁡N) additional one-qubit gates are sufficient to implement one segment e^−iCt/*r*^; and the total complexity to implement *r* segments will be *O*(*tK*) calls of controlled-*O*_*c*_ and its inverse as well as O(tKlog⁡N) additional one-qubit gates, where K=O(log⁡(t/ϵ)/loglog⁡(t/ϵ)). ▪

Note that we assumed the spectral norm ∥*C*∥=1. To explicitly put it in the complexity in theorem 4.1, we can simply replace *t* by ∥*C*∥*t*.

## 5. Inverse of circulant matrices

Following from §4, we now show that the HHL algorithm can be extended to solve systems of circulant matrix linear equations. We assume *C* to be Hermitian in this section in order for the phase estimation procedure to work.

**Theorem 5.1 (Inverse of circulant matrices).**

*There exists an algorithm creating the quantum state C*^−1^|*ψ*〉/∥*C*^−1^|*ψ*〉∥ *within error ϵ given an arbitrary quantum state* |*ψ*〉, *using* $\tilde{O}(\kappa^{2}/\epsilon )$ *calls of controlled-O*_*c*_ *and its inverse, O*(*κ*) *calls of O*_*ψ*_*, as well as* $\tilde{O}(\kappa^{2}\log N/\epsilon )$ *additional one- and two-qubit gates.*^2^

**Proof.**

The basic procedure is the same as the HHL algorithm [9], except that *C* is a dense circulant matrix rather than sparse as required by the HHL algorithm, which is summarized below.

1. Apply the oracle *O*_*ψ*_ to create the input quantum state |*ψ*〉:
  $$\left|0^{L}\right\rangle \overset{O_{\psi }}{\to }\left|\psi \right\rangle =\sum_{j=0}^{N-1}b_{j}\left|u_{j}\right\rangle ,$$
  where ${\left\{\left|u_{j}\right\rangle \right\}}_{j=0}^{N-1}$ are the eigenvectors of *C*.
2. Run phase estimation of the unitary operator e^i2*πC*^:
  $$\sum_{j=0}^{N-1}b_{j}\left|u_{j}\right\rangle \to \sum_{j=0}^{N-1}b_{j}\left|u_{j}\right\rangle \left|\Lambda_{j}\right\rangle ,$$
  where *Λ*_*j*_ are the eigenvalues of *C* and *Λ*_*j*_≤1.
3. Perform a controlled-rotation on an ancillary qubit:
  $$\sum_{j=0}^{N-1}b_{j}\left|u_{j}\right\rangle \left|\Lambda_{j}\right\rangle \left|0\right\rangle \to \sum_{j=0}^{N-1}b_{j}\left|u_{j}\right\rangle \left|\Lambda_{j}\right\rangle \left(\frac{1}{(\kappa \Lambda_{j})\left|1\right\rangle }+\sqrt{1-\frac{1}{\left(\kappa^{2}\Lambda_{j}^{2}\right)}}\left|0\right\rangle \right),$$
  where *κ* is the condition number defined in §2 to make sure that 1/(*κΛ*_*j*_)≤1 for all *j*. The realization of this controlled-rotation requires the computation of $\Lambda_{j}^{-1}$’s [43].
4. Undo the phase estimation and then measure the ancillary qubit. Conditioned on getting 1, we have an output state $\propto \sum_{j=0}^{N-1}b_{j}/\Lambda_{j}\left|u_{j}\right\rangle$ and the success rate $p=\sum_{j=0}^{N-1}|b_{j}/\kappa \Lambda_{j}{|}^{2}=\Omega (1/\kappa^{2})$.

Error occurs in step 2 in Hamiltonian simulation and phase estimation. The complexity scales sublogarithmically with the inverse of error in Hamiltonian simulation as in theorem 4.1 and scales linearly with it in phase estimation [1]. The dominant source of error is phase estimation. Following from the error analysis in [9], a precision *O*(*ϵ*/*κ*) in phase estimation results in a final error *ϵ*. Taking the success rate *p*=*Ω*(1/*κ*^2^) into consideration, the total complexity would be $\tilde{O}(\kappa^{2}/\epsilon )$, with the help of amplitude amplification [28]. ▪

For *s*-sparse Hamiltonians (with at most *s* non-zero entries in any row or column), the HHL algorithm scales as $\tilde{O}(s^{2}\kappa^{2}\log N/\epsilon )$ [9]. In this work, we extended the HHL procedure to dense Hamiltonians with special structure and proved the scaling is independent of matrix sparsity. This simplification stems from the efficient implementation of select(*V*) which makes possible the decomposition of *C* into *O*(*N*) terms without introducing *O*(*N*) into the computational complexity.

## 6. Products of circulant matrices

Products of circulant matrices are also circulant matrices, because a circulant matrix can be decomposed into a linear combination of ${\left\{V_{j}\right\}}_{j=0}^{N-1}$ that constitute a cyclic group of order *N* (we have *V*_*j*_*V*_*k*_=*V*_(*j*+*k*) mod  *N*_). Suppose *C*^(1,2)^=*C*^(1)^*C*^(2)^ is the product of two circulant matrices *C*^(1)^ and *C*^(2)^ which have a parameter vector ***c***^(1,2)^, where

**Figure 5.**
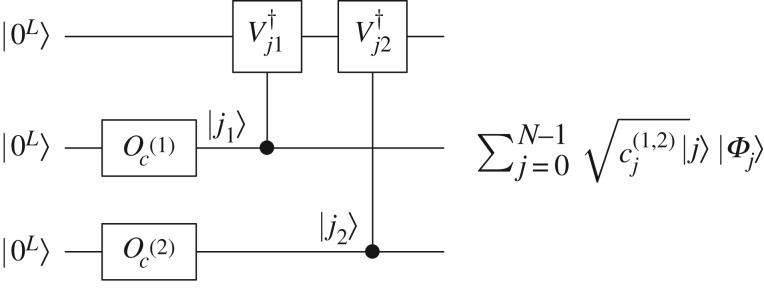
The quantum circuit of *O*_*c*^(1,2)^_. Here $V_{j}^{†}=\sum_{k=0}^{N-1}\left|(k+j)\;\mathrm{mod}\;\;N\right\rangle \left\langle k\right|$ and controlled-$V_{j}^{†}$ is a quantum adder.

6.1 $$c_{j}^{\left(1,2\right)}=\sum_{\begin{array}{c} j_{1},j_{2}\\ j_{1}+j_{2}\equiv j\;\;\mathrm{mod}\;\;N \end{array}}c_{j_{1}}^{\left(1\right)}c_{j_{2}}^{\left(2\right)},$$

where ***c***^(1)^ and ***c***^(2)^ are each the parameters of *C*^(1)^ and *C*^(2)^. Clearly, when the spectral norm of *C*^(1)^ and *C*^(2)^ are one, the spectral norm of *C*^(1,2)^ is also one. Classically, to calculate the parameters ***c***^(1,2)^ would take up *O*(*N*) space. However, in the quantum case, we will show that *O*_*c*^(1,2)^_, encoding ***c***^(1,2)^, can be prepared using one *O*_*c*^(1)^_ and one *O*_*c*^(2)^_. It means that the oracle for a product of circulant matrices can be efficiently prepared when its factor circulants are efficiently implementable, as illustrated in figure 5.

**Theorem 6.1 (Products of circulant matrices).**

*There exists an algorithm creating the oracle O*_*c*^(1,2)^_*, which satisfies*

6.2 $$O_{c^{\left(1,2\right)}}\left|0^{3L}\right\rangle =\sum_{j=0}^{N-1}\sqrt{c_{j}^{\left(1,2\right)}}\left|j\right\rangle \left|\Phi_{j}\right\rangle ,$$

*where* |*Φ*_*j*_〉 *is a unit quantum state dependent on j, using one O*_*c*^(1)^_*, one O*_*c*^(2)^_ *and* O(log⁡N) *additional one- and two-qubit gates.*

**Proof.**

We need 2*L* ancillary qubits divided into two registers to construct the oracle for the product of two circulant matrices. We start by applying *O*_*c*^(1)^_ and *O*_*c*^(2)^_ on the last 2 registers, we obtain

6.3 $$\left|0^{3L}\right\rangle \to \left|0^{L}\right\rangle \left(\sum_{j_{1}=0}^{N-1}\sqrt{c_{j_{1}}^{\left(1\right)}}\left|j_{1}\right\rangle \right)\left(\sum_{j_{2}=0}^{N-1}\sqrt{c_{j_{2}}^{\left(2\right)}}\left|j_{2}\right\rangle \right).$$

In order to encode $c_{j}^{\left(1,2\right)}$ in the quantum amplitudes, we once again apply quantum adders to achieve our goals. By performing the following transformation:

6.4 $$\left|0\right\rangle \left|j_{1}\right\rangle \left|j_{2}\right\rangle \to \left|j\right\rangle \left|j_{1}\right\rangle \left|j_{2}\right\rangle ,$$

where *j*≡( *j*_1_+*j*_2_) mod  *N*. This can be achieved using two quantum adders, we obtain the state

6.5 $$\sum_{j=0}^{N-1}\sqrt{c_{j}^{\left(1,2\right)}}\left|j\right\rangle \left|\Phi_{j}\right\rangle ,$$

because the amplitude of |*j*〉 is equal to $\sqrt{\sum_{\begin{array}{c} j_{1},j_{2}\\ j_{1}+j_{2}\equiv j\;\mathrm{mod}\;\;N \end{array}}{\left(\sqrt{c_{j_{1}}c_{j_{2}}}\right)}^{2}}=\sqrt{c_{j}^{\left(1,2\right)}}$. ▪

This algorithm can be easily extended to implementing oracles for products of *d* circulants, in which *d* oracles of factor circulants and *dL* ancillary qubits are needed. Though the oracle described in theorem 6.1 may not be useful in all quantum algorithms, owing to the additional |*Φ*_*j*_〉 in equation (6.2), it is applicable in §§2 and 4 according to lemma 6.2 (the generalized form of lemma 2.1) described below. It implies that this technique could also be useful in other algorithms related to circulant matrices.

**Lemma 6.2.**

*Let* $M=\sum_{\alpha_{j}}\alpha_{j}W_{j}$ *be a linear combination of unitaries W*_*j*_ *with α*_*j*_≥0 *for all j and* $\sum_{j}\alpha_{j}=1$. *Let O*_*α*_ *be any operator that satisfies* $O_{\alpha }\left|0^{m}\right\rangle =\sum_{j}\sqrt{\alpha_{j}}\left|j\right\rangle \left|\Phi_{j}\right\rangle$ (*m is the number of qubits used to represent* |*j*〉|*Φ*_*j*_〉) *and* $\mathrm{select}(W)=\sum_{j}\left|j\right\rangle \left\langle j\right|⊗I⊗W_{j}$. *Then*

6.6 $$(O_{\alpha }^{†}⊗I)\;\mathrm{select}(W)(O_{\alpha }⊗I)\left|0^{m}\right\rangle \left|\psi \right\rangle =\left|0^{m}\right\rangle M\left|\psi \right\rangle +\left|\Psi^{⊥}\right\rangle ,$$

*where* (|0^*m*^〉〈0^*m*^|⊗*I*)|*Ψ*^⊥^〉=0.

**Proof.**

aaaaa

$$\begin{array}{rl} (O_{\alpha }^{†}⊗I)\mathrm{select}(W)(O_{\alpha }⊗I)\left|0^{m}\right\rangle \left|\psi \right\rangle & =(O_{\alpha }^{†}⊗I)\mathrm{select}(W)\sum_{j}\sqrt{\alpha_{j}}\left|j\right\rangle \left|\Phi_{j}\right\rangle \left|\psi \right\rangle \\ & =(O_{\alpha }^{†}⊗I)\sum_{j}\sqrt{\alpha_{j}}\left|j\right\rangle \left|\Phi_{j}\right\rangle W_{j}\left|\psi \right\rangle \\ (\left|0^{m}\right\rangle \left\langle 0^{m}\right|O_{\alpha }^{†}⊗I)\sum_{j}\sqrt{\alpha_{j}}\left|j\right\rangle \left|\Phi_{j}\right\rangle W_{j}\left|\psi \right\rangle & =\left|0^{m}\right\rangle \sum_{j^{\prime }}\sqrt{\alpha_{j^{\prime }}}\left\langle j^{\prime }\right|\left\langle \Phi_{j^{\prime }}\right|\sum_{j}\sqrt{\alpha_{j}}\left|j\right\rangle \left|\Phi_{j}\right\rangle W_{j}\left|\psi \right\rangle \\ & =\left|0^{m}\right\rangle \sum_{j}\alpha_{j}W_{j}\left|\psi \right\rangle =\left|0^{m}\right\rangle M\left|\psi \right\rangle . \end{array}$$

 ▪

## 7. Application: solving cyclic systems

Vibration analysis of mechanical structures with cyclic symmetry has been a subject of considerable studies in acoustics and mechanical engineering [14,17]. Here we provide an example where the above proposed quantum scheme can outperform classical algorithms in solving the equation of motion for vibrating and rotating systems with certain cyclic symmetry.

**Figure 6.**
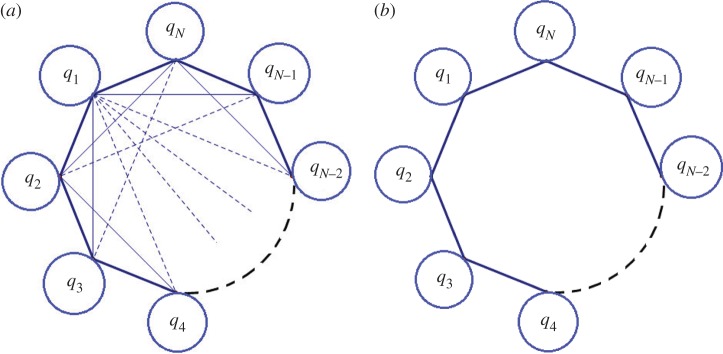
Topology diagram of an *N*-sector cyclic system. (*a*) A general cyclic system with coupling between any two sectors which can be solved using theorem 5.1. (*b*) A cyclic system with nearest-neighbour coupling which can be solved using the HHL algorithm [9].

The equation of motion for a cyclically symmetric system consisting of *N* identical sectors, as shown in figure 6, can be written as

7.1 $$M\ddot{q}+D\dot{q}+Kq=f,$$

where ***q*** and ***f*** are *N*-dimensional vectors, denoting the displacement of and the external force acting on each individual sector, respectively. The mass, damping and stiffness matrices are all circulants, represented by *M*=circ(*m*_1_,*m*_2_,…,*m*_*N*_), *D*=circ(*d*_1_,*d*_2_,…,*d*_*N*_) and *K*=circ(*s*_1_,*s*_2_,…,*s*_*N*_).

Assume all sectors have the same mass (*M*∝*I*) and there is zero damping (*D*=0). If the system is under the so-called travelling wave engine order excitation, the equation of motion can be simplified as [14]:

7.2 $$\ddot{q}+Kq=f\;e^{in\Omega t},$$

where the travelling wave is characterized by *f*_*j*_=*f* e^i2*πnj*/*N*^ for the external force vector ***f***, *n* is the order of excitation and *Ω* is the angular frequency of the excitation. We search for solutions of the form ***q***=***q*_0_** *e*^*inΩt*^, which leads to

7.3 $$(K-n^{2}\Omega^{2}I)q_{0}=f.$$

Since *K*−*n*^2^*Ω*^2^*I* is a circulant matrix, we can use theorem 5.1 to calculate

$$q_{0}={\left(K-n^{2}\Omega^{2}I\right)}^{-1}f.$$

It is important to consider the conditions under which theorem 5.1 works.

1. *K*−*n*^2^*Ω*^2^*I* is Hermitian. This is generally true for symmetric cyclic systems, where the coupling between *q*_*j*_ and *q*_*j*+*d*_ and the coupling between *q*_*j*_ and *q*_*j*−*d*_ are physically the same for any sector *j* and distance *d*.
2. *K*−*n*^2^*Ω*^2^*I* has non-negative (or non-positive) entries. Although this is not in general true, theorem 5.1 will work under a slight modification. We observe that the off-diagonal elements of *K*−*n*^2^*Ω*^2^*I* are always negative because the coupling force between two connecting sectors is always in the opposite direction to their relative motion.
  — If the diagonal elements of *K*−*n*^2^*Ω*^2^*I* are also negative, then no modification to the proposed procedure is necessary.
  — If the diagonal elements of *K*−*n*^2^*Ω*^2^*I* are positive, using the technique stated in §(a), we can simply replace select(*V*) with Ref_0_⋅select(*V*), where Ref_0_=|0^*L*^〉〈0^*L*^|−2*I* is a reflection operator operating on the first register.
3. The condition number *κ* of *K*−*n*^2^*Ω*^2^*I* is small. This is true when the couplings among sectors are relatively weak—when |*K*_0_−*n*^2^*Ω*^2^|≫*K*_1_ where *K*_0_ characterizes the coupling between a sector and the exterior and *K*_1_ characterizes the coupling among sectors.
4. The corresponding oracle *O*_*c*_ of the circulant matrix *K*−*n*^2^*Ω*^2^*I* can be efficiently implemented. It requires either there is a special structure of *K*−*n*^2^*Ω*^2^*I* or the information of *K*−*n*^2^*Ω*^2^*I* is stored in a qRAM in advance.

If all four conditions are satisfied, we have an exponential speed-up compared to classical computation. Note that the output ***q*_0_** is stored in quantum amplitudes, which cannot be read out directly. However, further computation steps can efficiently provide practically useful information about the system from the vector ***q*_0_**, for example the expectation value ***q*_0_**^†^*M****q*_0_** for some linear operator *M* or the similarity between two cyclic systems 〈*q*′_0_|*q*_0_〉 [9]. This type of speed-up is not achievable classically for it takes at least *O*(*N*) steps to read out the value of ***q*_0_**. It is also worth noting that the proposed algorithm, in contrast to previous quantum algorithms [3–7,9,35], works for dense matrices *K*−*n*^2^*Ω*^2^*I*. It means that the cyclic systems need not be subject to nearest-neighbour coupling.

## 8. Conclusion

In this paper, we present efficient quantum algorithms for implementing circulant (as well as Toeplitz and Hankel) matrices and block circulant matrices with special structures, which are not necessarily sparse or unitary. These matrices have practically significant applications in physics, mathematics and engineering-related fields. The proposed algorithms provide exponential speed-up over classical algorithms, requiring fewer resources (2log⁡N qubits) and having lower complexity (O(log⁡N/∥C|ψ⟩∥)) in comparison with existing quantum algorithms. Consequently, they perform better in quantum computing and are more feasible to experimental realization with current technology. Obstacles still exist, though, in the efficient realization of the oracles to generate the components of the circulant matrices.

Besides the implementation of circulant matrices, we discover that we can perform the HHL algorithm on circulant matrices to implement the inverse of circulant matrices, by adopting the Taylor series approach to efficiently simulate circulant Hamiltonians. Owing to the special structure of circulant matrices, we prove that they are one of the types of the dense matrices that can be efficiently simulated. Being able to implement the inverse of circulant matrices opens a door to solving a variety of real-world problems, for example, solving cyclic systems in vibration analysis. Finally, we show that it is possible to construct oracles for products of circulant matrices using the oracles for their factor circulants, a technique that will be useful in related algorithms.

## Footnotes

1 By controlled-*O*_*c*_, we mean the operation |0〉〈0|⊗*I*+|1〉〈1|⊗*O*_*c*_.

2 We use the symbol $\tilde{O}$ to suppress polylogarithmic factors.

## Data accessibility

This paper does not include further supporting information.
